# Breaking the Silence: The Effectiveness of a Bystander Intervention Program Against Dating Violence

**DOI:** 10.1192/j.eurpsy.2026.12044

**Published:** 2026-07-31

**Authors:** S. Er, S. Buzlu

**Affiliations:** 1 Institute of Graduate Studies; 2 Florence Nightingale Faculty of Nursing, Istanbul University-Cerrahpaşa, Istanbul, Türkiye

## Abstract

**Introduction:**

Dating violence (DV)—marked by coercion and control—has been associated with physical injury, depression, reduced academic performance, and substance use among university students. Bystander-focused prevention has gained prominence internationally; however, no culturally adapted program for university students in Türkiye has been evaluated.

**Objectives:**

This randomized, controlled, repeated-measures (pretest–posttest–follow-up) experimental study was conducted to evaluate the effectiveness of a bystander intervention program to prevent DV among nursing students.

**Methods:**

The study was conducted among second-year nursing students at a public university in Istanbul (N=83; mean age 19.99±1.15; 79.5% women; intervention n=42; control n=41). Data were collected between December 2024 and March 2025 using a Personal Information Form, the Bystander Efficacy Scale, the Intent to Help Scale–Brief Version, and the Bystander Behaviour Scale (For Friends). The “Breaking the Silence: Bystander Intervention Program against Dating Violence” was developed by the investigators following a comprehensive literature review and relevant evidence-based guidelines and was culturally adapted to the Turkish university context. The intervention was delivered as a five-session, small-group workshop. Its theoretical framework drew on the Decision-Making Model of Bystander Intervention and the Theory of Planned Behavior. Distinct from existing programs, one session addressed bystander responses to online DV and another focused on bystander self-care. Group differences were analysed with χ² tests, t-tests, and repeated-measures ANOVA with Bonferroni corrections. The study was conducted and reported in accordance with the CONSORT (Consolidated Standards of Reporting Trials) guidelines, and it was registered and assigned a ClinicalTrials.gov identifier (NCT06178016).

**Results:**

Compared with controls, the intervention group demonstrated significant gains from pretest to posttest that were sustained at follow-up in bystander efficacy (p<0.001), intent to help both friends and strangers (p<0.001), and total bystander behaviour, driven by increases in proactive behaviour (p<0.001). Effects at follow-up showed partial attenuation from posttest but remained above baseline for bystander efficacy and intent to help.

**Conclusions:**

A culturally tailored bystander intervention for university students significantly improved efficacy, intentions, and proactive behaviours to address DV. This research can encourage the development of new bystander intervention-based programs on different university campuses to prevent DV among university students. In addition, the findings of this study underscored the need for culturally tailored bystander programs addressing diverse forms of violence and bullying in Türkiye.

**Disclosure of Interest:**

S. Er Grant / Research support from: This doctoral thesis was supported by the Scientific and Technological Research Council of Türkiye (TÜBİTAK), Project No. 324K026., S. Buzlu Consultant of: Prof. Dr. Sevim Buzlu was the supervisor of this doctoral thesis.

